# The satisfaction with radiology residency training in China: results of a nationwide survey

**DOI:** 10.1186/s13244-022-01329-x

**Published:** 2022-12-15

**Authors:** Peicheng Wang, Jingfeng Zhang, Yanhua Chen, Yanrong He, Lijun Shen, Maoqing Jiang, Zhenghan Yang, Jianjun Zheng, Zhenchang Wang, Jiming Zhu

**Affiliations:** 1 Vanke School of Public Health, Tsinghua University, Haidian District, Beijing, 100084 China; 2 School of Medicine, Tsinghua University, Beijing, China; 3 Department of Radiology, Hwa Mei Hospital, University of Chinese Academy of Sciences, Ningbo, China; 4 Department of Radiology, Beijing Friendship Hospital, Capital Medical University, Beijing, China; 5 Institute for Healthy China, Tsinghua University, Beijing, China

**Keywords:** Radiology resident, Standardized residency training, Satisfaction, China

## Abstract

**Background:**

National data on the satisfaction of radiology residents enrolled in standardized residency training (SRT) are rather scarce in China. This study identified a set of potential factors concerning SRT satisfaction among radiology residents as well as the association between SRT satisfaction and post-competency and well-being.

**Methods:**

A total of 3666 radiology residents who were receiving SRT during 2020 in China were recruited across all 31 provinces. The cumulative odds logistic regression was used to examine the potential factors associated with SRT satisfaction as well as associations between satisfaction and well-being, burnout, professional identity and competency.

**Results:**

The prevalence of satisfaction with SRT was 68.6%. Participants who were male, worked in central China, aged more than 28 years old, had long working hours and claimed increased workload during the COVID-19 pandemic were more likely to be unsatisfied with the SRT program. Participants who were more satisfied with the radiation protection were more likely to report higher degree of SRT satisfaction (OR = 3.00, 95% CI 2.58–3.50). In addition, SRT satisfaction was positively associated with well-being, professional identity, competency and lower burnout.

**Conclusions:**

Perceived satisfaction can be introduced into hospital management, as it may reflect the overall situation of the residents during residency training and influence radiologists’ well-being, professional identification and competency. Appropriate measures should be taken to reduce the risk of radiation exposure, ensure employee safety (such as risk assessment system and paid time off), provide radiology residents with fair treatment and guarantee the time out of working for optimizing their professional ability.

**Supplementary Information:**

The online version contains supplementary material available at 10.1186/s13244-022-01329-x.

## Key points

- The radiology residents at all levels in China were systematically landscaped.
- 68.6% of the 3666 residents reported satisfaction with the residency training.
- Radiation protection was significantly associated with residency satisfaction.
- Satisfaction significantly impacts radiology residents’ well-being, professional identity and self-reported competency.
- The findings provide new insights into sustaining and improving the radiology residency.

**Supplementary Information:**

The online version contains supplementary material available at 10.1186/s13244-022-01329-x.

## Introduction

Radiology, as one of the essential parts of modern patient care, includes many subspecialties such as radiation diagnosis, imaging technology, ultrasonic diagnosis, nuclear medicine and interventional radiology [1]. The specialty of radiology covers all levels of health care delivery, virtually all medical specialties and all ages of patients [1, 2], which brings many challenges and troubles to radiologists. For example, patients’ needs and the COVID-19 pandemic require increasing radiologists skill and specialization [3, 4]; the artificial intelligence increases consistency in image analysis [2] but may revolutionize radiologists’ workflows [5], the insufficient interaction with clinical colleagues and the lower social status of radiologists [3, 6]. Previous meta-analysis studies demonstrated that radiologists had a higher burnout rate (77.16%) and were the least happy group when compared to residents in other fields [7–9]. Therefore, it is urgent to focus on training program and building the resilience of radiologist.

The standardized residency training (SRT) is a crucial step in the development of the competent and resilient radiologists. In 2013, China rolled out a compulsory residency program nationwide. Doctors from 36 specialties including radiology [10, 11] were required to participate in a 3-year training. As a compulsory postgraduate program, SRT extends the length of formal training. International experience suggests that residency system plays a significant role in cultivating competent medical doctors [12]. According to the content and standards of China’s radiology SRT, radiology residents should rotate through core departments and produce a certain number of diagnostic radiology imaging reports (e.g., CT interpretation and reporting ≥ 4500 cases, MR interpretation and reporting ≥ 1800 cases, radiograph interpretation and reporting ≥ 1600 cases and interventional procedures ≥ 20 cases). What’s more, they are expected to supervise medical students/junior residents and conduct scientific research. Upon completion of the three-year SRT, they are required to independently carry out clinical practice of diagnostic radiology for common and even some intractable illnesses and conditions; as physicians, they are also expected to acquire essential medical emergency skills. At the end of June 2021, 557 radiology residency programs had been established in China, covering 7851 residents and 4357 professional master student nationwide. Despite the great achievements, there is still a need for continuous improvement in radiology SRT. As demonstrated by our previous study, China’s SRT should further strengthen some “soft” content (e.g., communication skills) to improve the train quality [10].

In order to improve the quality of training, it is important to establish or improve residency training based on resident feedback (e.g., training program satisfaction survey). Resident satisfaction is often cited as an important indicator of SRT program quality [13, 14]. Some studies showed that dissatisfaction with residency training might cause anxiety and burnout among trainees and affect their professional performance [7, 15–17]. In contrast, being satisfied with the residency program would potentially improve productivity as well as the retention rate for general surgery programs [18, 19]. So far, most of the extant studies focus on the satisfaction of resident doctors in ophthalmology, surgery, internal medicine and nursing [15, 19, 20]. Related factors such as clinical supervision and support, working hours, income, work-life balance and study time were all considered related to the development of dissatisfaction [19, 21, 22]. However, considering the difference of work orientation and job content among different specialties, the associated factors influencing radiologist satisfaction may be different from those of other specialties [21, 22]. An evaluation of the SRT program satisfaction in radiology is needed to get a holistic understanding of the program and to motivate policy departments to launch targeted interventions for improving the training quality of the program.

China’s central government has asserted that “it is significant to improve the standardized training policy for residents, to guarantee the remuneration of residents, and to strengthen their sense of gain” [23]. Moreover, SRT program requires trainees to constantly learn new skills and acquire new knowledge to meet the growing demand for physicians, especially in the post-COVID-19 era [3]. This nationwide survey aims to examine radiology residents’ satisfaction with the SRT program and potential factors associated with dissatisfaction (e.g., radiation protection) and further clarify the importance of satisfaction in shaping job competency and well-being. This study would help establish a better SRT educational system and aims to provide valuable insights for other countries to develop their own residency training programs.

## Methods

### Study design and participants

This national survey was conducted by the Chinese Association of Radiologists (CAR) during December 1, 2020, to April 30, 2021. Residency training-related information was collected from radiology residents (participants were identified and recruited by the CAR) during their residency training across 31 provinces (data on Macao, Hong Kong and Taiwan are not available) in China. Questionnaires were distributed via a widely used online survey tool called “Wenjuanxing” in China. After obtaining their informed consent, respondents were required to complete an online questionnaire anonymously. They were allowed to withdraw at any time without penalty. A total of 3666 out of 12,208 potentially eligible radiologists who are receiving SRT during 2020 in China responded effectively to our survey, yielding an overall effective response rate of 30.0%. 407 (73.1%) of 557 radiology programs were covered.

### Study outcomes and measures

#### Radiology standardized residency training satisfaction (radiology SRT satisfaction)

Radiology SRT satisfaction was asked by “Overall, you are satisfied with the standardized residents training program” [13, 24]. Different degrees of satisfaction were defined according to a 7-point Likert scale: strongly disagree—1, disagree—2, less disagree—3, moderate—4, less agree—5, agree—6, strongly agree—7. Participants with a score ≥ 5 (median score = 5) were defined as being satisfied with their residents training program in general. (We coded the dissatisfaction as 0 and satisfaction as 1.)

#### Common factors of job dissatisfaction

Common dissatisfaction factors among radiology residents were identified by the question: “What makes you dissatisfied with your current job?” with response options: “low income,” “high workload,” “contentious doctor-patient relationships,” “complex rules and regulations” and “low social status” [25]. They can choose at least one from these five options.

*Radiation protection satisfaction.* Radiation protection satisfaction was measured using a 5-point Likert scale (strongly dissatisfied—1, dissatisfied—2, moderate—3, satisfied—4, and strongly satisfied—5) by asking: “Are you satisfied with the radiation protection in your current job?”. Participants with a score ≥ 4 (median score = 4) were defined as being satisfied with the radiation protection.

#### Competency of radiology residents

Milestone-based competency assessment for residency is one of the common requirements of the Accreditation Council for Graduate Medical Education (ACGME) [10]. We adopted the conceptual framework of the ACGME Six Core Competencies and go into the 9 detailed milestones from 24 items diagnostic radiology milestones. Milestones are arranged from Level 1 to Level 5, and Level 4 is designed as a graduation goal but does not represent a graduation requirement. Five levels were responded on a 9-point scale (total score ranges from 9 to 81): Level 1–1, between Level 1 and Level 2–2, Level 2–3, between Level 2 and Level 3–4, Level 3–5, between Level 3 and Level 4–6, Level 4–7, between Level 4 and Level 5–8, Level 5–9 (an example set of milestones for one sub-competency is shown in Additional file 1: Table S1) [26]. Participants with a total score ≥ 26 (median score = 26) were defined as being competent in the corresponding item of their residents training.

#### Socio-demographic characteristics

Socio-demographic variables include gender (male and female), age (≤ 28 years, and > 28 years), marital status (unmarried and married), educational level (bachelor’s degree and master’s or doctoral degree), training years (the 1st year, the 2nd year and the 3rd year), region (East, Middle, West and Northeast), annual after-tax income (≤ 10,000, 10,000–40,000, 40,000–60,000 and > 60,000, in RMB), working hours per week (≤ 40, 41–48 and > 48 h) and workload changes (a decrease, no change and an increase) during the COVID-19 pandemic (January to May 2020, when the whole country was affected).

### Statistical analysis

The descriptive analysis of continuous variables with normal distribution was calculated using means and standard deviations, while continuous variables with skewed distribution were calculated using medians and interquartile ranges. The percentage of SRT satisfaction and radiation exposure protection satisfaction among participants were reported, and SRT satisfaction between females and males was compared using chi-square tests.

Logistic regression model was constructed to explore potential factors associated with the SRT satisfaction. Candidate factors include the socio-demographics and radiation protection satisfaction. To clarify the importance of SRT satisfaction, a new set of ordinal logistic regression models was constructed where burnout, well-being, professional identity and post-competency were dependent variables and the SRT satisfaction was the key explanatory variable. A variance inflation factor test was conducted to detect multicollinearity among independent variables. Statistical analyses were performed using SAS 9.4 (SAS Institute, Cary, NC, USA), and significance was defined at the 0.05 level, with 2-tailed t tests. For all regression results, ORs and 95% CIs were reported. For four variables with missing values, Markov chain Monte Carlo (MCMC) method of multiple imputation was used for imputing missing values and combining inferences. Detailed information on missing rates of each variable and strategies for management of missing data are presented in Additional file 1: Table S2.

## Results

### Characteristics of study participants

Table 1 shows that 58% of the respondents were female. 73.3% aged 28 and younger, 76.0% unmarried and 92.1% of them holding a bachelor’s degree. Over one-third of the respondents were from eastern region (40.5%) or western regions (33.3%).

**Table 1:** Characteristics of surveyed participants in China

Variables	Total (n = 3666)
*Region*	
East	1486 (40.5)
Central	742 (20.2)
West	1220 (33.3)
Northeast	218 (6.0)
*Age (years), Mean (SD)*	27.3 (2.6)
≤ 28	2687 (73.3)
> 28	979 (26.7)
*Gender*	
Male	1539 (42.0)
Female	2127 (58.0)
*Training years*	
The 1st year	1285 (35.1)
The 2nd year	1179 (32.2)
The 3rd year	1202 (32.8)
*Degree*	
Bachelor	3375 (92.1)
Master or doctoral	291 (7.9)
*Marital status*	
Unmarried	2829 (77.2)
Married or others	837 (22.8)
Annual after-tax income (RMB), Median (IQR)	35,000 (6000–60,000)
≤ 10,000	1253 (34.2)
10,001–40,000	880 (24.0)
40,001–60,000	737 (20.1)
> 60,000	796 (21.7)
*Working hours per week (hours), Mean (SD)*	44.0 (12.1)
≤ 40	2065 (56.3)
41–48	801 (21.9)
> 48	800 (21.8)
*Workload changes during the COVID-19 pandemic*	
Decrease	1296 (35.4)
No change	1457 (39.7)
Increase	913 (24.9)

^a^Ratio of US dollar to Chinese Yuan (RMB) = 6.8974 in 2020 year

Only 21.7% of the respondents indicated an annual after-tax income of 60,000 RMB or more (about $ 8698.9). Regarding working hours per week, the largest proportion of respondents (56.3%) worked less than 40 h per week and 21.8% of the participants worked more than 48 h per week. Nearly a quarter (24.9%) of the participants reported an increased workload during the COVID-19 pandemic (Table1).

### Prevalence of radiology SRT program satisfaction

Table 2 shows the data on SRT program satisfaction. The mean SRT satisfaction score was 5.15 with a standard deviation of 1.33. 68.6% expressed satisfaction with SRT program. Moreover, females in our sample had a higher rate of satisfaction (70.2%) compared with males (66.3%, *p* < 0.05).

**Table 2:** Prevalence of SRT satisfaction in a sample of radiology residents in China

	Total	Gender		*p* value
		Male	Female	
*SRT satisfaction scores, mean (SD)*	5.15 (1.33)	5.08 (1.46)	5.21 (1.23)	0.005
Satisfaction, n (%)	2514 (68.6)	1020 (66.3)	1494 (70.2)	0.011
Dissatisfaction	1152 (31.4)	519 (33.7)	633 (29.8)	
*Common areas for dissatisfaction, n (%)*				
Low income	2529 (69.0)	1152 (74.8)	1377 (64.7)	< 0.001
High workload	1542 (42.1)	677 (44.0)	865 (40.7)	0.044
Contentious doctor-patient relationships	1109 (30.3)	487 (31.6)	622 (29.2)	0.118
Complex rules and regulations	969 (26.4)	469 (30.5)	500 (23.5)	< 0.001
Low social status	497 (13.6)	272 (17.7)	225 (10.6)	< 0.001
*Radiation protection, mean (SD)*	3.79 (0.74)	3.83 (0.77)	3.75 (0.72)	0.001
Satisfaction, n (%)	2509 (68.4)	1085 (70.5)	1424 (67.0)	0.022
Dissatisfaction	1157 (31.6)	454 (29.5)	703 (33.0)	

The top three reasons for dissatisfaction during the training were low income (69.0%), high workload (42.1%) and contentious doctor–patient relationships. Besides, males were more likely to be dissatisfied with low income, high workload, complex rules and regulations and low social status of their job compared to females (*p* < 0.05).

We also examined the satisfaction for radiation protection among residents during the SRT program, 68.4% of respondents were satisfied with their radiation protection in general, and females reported a lower rate of radiation protection satisfaction (67.0%) than males (70.5%, *p* < 0.05) (Table 2).

### Factors associated with SRT satisfaction

We examined the associations between socio-demographic characteristics and SRT satisfaction in Model 1. The results showed that working in the central areas of China (OR = 0.79, 95% CI 0.65–0.96, *p* = 0.017), the higher age-group (age > 28 years, OR = 0.80, 95% CI = 0.66–0.97, *p* = 0.022), being male (female, OR = 1.21, 95% CI 1.05–1.40, *p* = 0.009), senior SRT years (the 2nd year, OR = 0.71, 95% CI 0.59–0.85; the 3rd year, OR = 0.79, 95% CI 0.66–0.95, *p* < 0.05) and longer working hours (41 to 48 h per week, OR = 0.69, 95% CI 0.57–0.82; > 48 h per week, OR = 0.46, 95% CI 0.39–0.55, *p* < 0.001) significantly affect participants’ satisfaction with SRT program. Moreover, the increased workload ((OR = 0.72, 95% CI 0.60–0.86, *p* < 0.001) during the COVID-19 pandemic among radiology residents reduced their satisfaction with the SRT program.

After adjusting for participants’ characteristics and other factors in Model 2, participants who were more satisfied with the radiation protection were 3.00 (95% CI 2.58–3.50, *p* < 0.001) times more likely to report higher SRT satisfaction (Table 3).

**Table 3:** Effects of respondent characteristics on SRT satisfaction

Variables	Model 1	*p* value	Model 2	*p* value
	AOR (95%CI)		AOR (95%CI)	
*Radiation protection*				
Dissatisfaction	–	–	Ref	
Satisfaction	–	–	3.00 (2.58, 3.50)	< 0.001
*Hospital region*				
East	Ref		Ref	
Central	0.79 (0.65, 0.96)	0.017	0.90 (0.74, 1.10)	0.304
West	1.00 (0.84, 1.18)	0.994	1.04 (0.87, 1.24)	0.688
Northeast	1.14 (0.82, 1.57)	0.449	1.09 (0.78, 1.53)	0.619
*Age*				
≤ 28 years	Ref		Ref	
> 28 years	0.80 (0.66, 0.97)	0.022	0.82 (0.67, 0.99)	0.042
*Gender*				
Male	Ref		Ref	
Female	1.21 (1.05, 1.40)	0.009	1.27 (1.09, 1.47)	0.002
*Training years*				
The 1st year	Ref		Ref	
The 2nd year	0.71 (0.59, 0.85)	< 0.001	0.71 (0.59, 0.85)	< 0.001
the 3rd year	0.79 (0.66, 0.95)	0.013	0.79 (0.66, 0.96)	0.016
*Degree*				
Bachelor	Ref		Ref	
Master or doctoral	0.82 (0.63, 1.07)	0.146	0.87 (0.66, 1.14)	0.313
*Marital status*				
Married or others	Ref		Ref	
Unmarried	0.96 (0.79, 1.16)	0.667	0.98 (0.81, 1.20)	0.873
*Annual after-tax income*				
≤ 40,000	Ref		Ref	
> 40,000	1.17 (1.00, 1.37)	0.053	1.08 (0.92, 1.27)	0.363
*Working hours per week*				
≤ 40	Ref		Ref	
41–48	0.69 (0.57, 0.82)	< 0.001	0.68 (0.57, 0.82)	< 0.001
> 48	0.46 (0.39, 0.55)	< 0.001	0.45 (0.38, 0.54)	< 0.001
*Workload changes during the COVID-19*				
No change	Ref		Ref	
Decrease	0.93 (0.78, 1.10)	0.387	0.95 (0.79, 1.13)	0.553
Increase	0.72 (0.60, 0.86)	< 0.001	0.79 (0.65, 0.96)	0.015

### Association of SRT satisfaction with burnout, well-being, professional identity and post-competency

As shown in Table 4, SRT satisfaction was positively associated with well-being (OR = 9.74, 95% CI 8.14–11.65, *p* < 0.001) and negatively associated with burnout (OR = 0.25, 95% CI 0.20–0.30, *p* < 0.001) after adjusting for participants’ characteristic factors. Similarly, respondents with higher SRT satisfaction were significantly more likely to report stronger professional identity (OR = 14.03, 95% CI 11.60–16.97, *p* < 0.001) and competency (OR = 1.32, 95% CI 1.14–1.54, *p* < 0.001) (Table 4).

**Table 4:** Association of SRT satisfaction with well-being, professional identity and post-competency

	Greater well-being	Burnout	Professional identity	Competency
	AOR (95% CI)	AOR (95% CI)	AOR (95% CI)	AOR (95% CI)
*SRT satisfaction*				
Dissatisfaction	Ref	Ref	Ref	Ref
Satisfaction	9.74 (8.14, 11.65)***	0.25 (0.22, 0.30)***	14.03 (11.60, 16.97)***	1.32 (1.14, 1.54)***

Burnout was measured by the Maslach Burnout Inventory-Human Service Survey (MBI-HSS), and participants with high EE (≥ 27) and/or DP (≥ 10) scores were defined as “having burnout.” Greater well-being is a dummy variable indicating respondents who felt overall satisfied with their psychological, emotional and physical. Professional identity is a dummy variable indicating respondents had higher professional identity level

***p < 0.001. All models were controlled for participants’ characteristics

## Discussion

This study was the first nationwide survey that comprehensively investigated the SRT satisfaction among radiologists who were during their residency training in China. It was found that participants were moderately satisfied with radiology SRT. Common factors associated with SRT dissatisfaction include older age (age > 28 years), being male, longer SRT year and working hours per week and working in the central of China. In addition, the results showed that SRT satisfaction was closely related to the satisfaction with radiation protection. What’s more, there were positive associations between SRT satisfaction and less burnout, higher well-being, greater professional identity and job competency.

The prevalence of SRT satisfaction among radiology residents in China was 68.6%, which was higher than Poland (43.0%) [27] and lower than that of Rochester, Minnesota (91.6%) [28]. In addition to the regional differences across countries, individual factors in China should also be considered. For instance, male radiologists were more likely to be dissatisfied with SRT programs, which was inconsistent with previous findings conducted in Spain [13] and Saudi Arabia [15]. Reasons might be that males might face more stress in their lives and workplaces [29, 30]. In this study, the higher rate of being married (26.3% vs. 19.6%) and having children (15.8% vs. 11.1%) in males than in females may increase males’ life and training stress.

Personal income fails to explain the variation in SRT satisfaction in our study, which is inconsistent with previous studies [22, 25, 30, 31]. One possible reason was that radiology residents treat the SRT as more of a learning experience process rather than a job. Income was only one aspect when they measured the gains from the SRT. Knowledge acquisition, skill improvement and experience enrichment were also key components. Another possible explanation might be that the general dissatisfaction (69.0%) with the low income, especially given that their income was generally very low, did not necessarily affect the satisfaction with the SRT program. Indeed, the annual income for most radiology residents ($5074) was only about 0.45 times China’s per capita GDP in 2020 ($11,300) and much lower than the annual salaries ($8000-$75,000) of radiology residents in other countries [31].

Long working hours were another factor associated with trainees’ SRT dissatisfaction. Previous findings demonstrated that long working hours affect residents and even medical staff not only in terms of satisfaction, but also in terms of patient safety, physician burnout, turnover intention, quality of life, and so on [25, 30, 32, 33]. The respondents in our study worked an average of 44.0 h per week, and 21.5% of them worked on night shifts more than once a week. Therefore, it would be necessary to guarantee their personal disposable time, especially for work-life balance. Our study found that those who were more satisfied with SRT reported more time dealing with personal and family matters. In addition, other studies have shown that measures giving residents more time off to attend academic activities and engage in regular exercise contribute to trainee satisfaction [13, 21, 22, 32].

Consistent with earlier studies, residents in the first-year were more satisfied with SRT than in subsequent years [13, 34]. Main reasons may be due to changes in their attitude and mood. Fewer experience, more expectations and lower routine work appear to make junior residents more satisfied with the SRT program [8, 13, 35]. Given that teaching has currently become one of the core competencies to be achieved according to the ACGME [35–37], our study also provided evidence that SRT satisfaction was higher among residents who were involved in the management of the SRT program or were available to provide mentoring to medical students or junior residents. We suggest that SRT program director could involve senior residents more in the program management and teaching, thus providing more practical suggestions for program improvement.

Radiologists and radiologic technologists are one of the groups with the highest occupational exposure to low-dose radiation (e.g., medical imaging, computed tomography) [38, 39]. Although it was still unclear whether populations develop illness (e.g., cancer, leukemia) caused by low-dose radiation exposure, the risk of radiation to fetal and cataracts was clear [39, 40]. The association between radiation protection satisfaction and SRT satisfaction in radiology residents was also significant in the present study. In addition, females (67.0%) were less satisfied with radiation protection than males (70.5%). Therefore, effective radiation protection measures at the institutional level, such as establishing risk assessment system for radiation protection, should be taken [41]. Moreover, 2–4 weeks’ annual health leave for medical staff and special care of female radiologists during their pregnancy should be considered to minimize occupational exposure hazards.

The COVID-19 pandemic dramatically changed the way people lived and worked. Accordingly, the SRT program and the radiology trainees had also to adjust [42]. For instance, the implementation of physical distancing and the distance learning limited their on-site work [42, 43]; radiology residents were temporarily redeployed to clinical inpatient services (e.g., internal medicine wards and intensive care units) to care for COVID-19 patients [42, 44]. We found that the increased workload among radiology residents would reduce their satisfaction with the SRT program during the pandemic. Residents would likely encounter COVID-19 patients during their daytime work or were redeployed to be directly involved in the management of patients, which might influence their mental health or well-being [44, 45]. Another possible explanation was that the increased workload during the pandemic deprived residents of their time committed to clinical rotations or procedural practice. The consequent concerns for career development made them less satisfied with the SRT program [43–46].

Finally, our findings emphasized the importance of SRT satisfaction, as it may affect the well-being of radiology residents and improve their career commitment and clinical productivity. In line with previous studies [17, 47, 48], our study found that SRT satisfaction was significantly associated with lower burnout and higher level of well-being, professional identity and self-reported competency. Participants’ satisfaction was often used to assess the effectiveness of a medical training program and improve curriculum for enhancing the competence of trainees [47–49]. From the SRT satisfaction assessment, we can get first-hand information about the access to educational resource and quality of the medical training program.

## Limitation

A few limitations of this study should be acknowledged. First, our survey lacks assessments on the academic dedication (e.g., number of publications, participation in conferences, dissertations), on-call days and mentoring from the faculty, which may be associated with SRT satisfaction among radiology residents. Second, though all the SRT training sites are China’s top hospitals, our data collected do not allow us to discuss the impact of detailed institutional information (e.g., outpatient visits, patient beds, the hospital’s overall health workforce) on residents’ SRT satisfaction. Third, causal relationships cannot be inferred from cross-sectional observational data. Future studies could establish a 3–5 years’ follow-up and conduct a longitudinal assessment of radiology residents, so that we can identify additional factors in relation to SRT satisfaction and determine their professional evolution.

## Conclusions

In conclusion, based on a large national sample in China, we found an upper middle SRT satisfaction of radiology trainees. Perceived satisfaction is an effective tool to assess the overall quality of the medical residency programs and has a great impact on professional identification and training performance. Appropriate measures should be taken to enhance radiation protection (such as establishing risk assessment system for radiation protection, ensuring 2–4 weeks’ annual health leave for radiologists) and guarantee the paid time off, so as to improve the quality and satisfaction of SRT program.

## Supplementary Information

**Additional file 1**. An example of milestones for a sub-competency and the processing of missing data.

## Data Availability

The original data generated from this study and the analyzed results will be available from the corresponding author (jimingzhu@tsinghua.edu.cn) upon reasonable request and with permission of the Chinese Association of Radiologists.

## Abbreviations

ACGME: Accreditation Council for Graduate Medical Education
SRT: Standardized residency training
