# Supplementary material for: The satisfaction with radiology residency training in China: results of a nationwide survey
Source: Insights Imaging. 2022 Dec 15;13:196. doi: 10.1186/s13244-022-01329-x (PMC9753880; doi:10.1186/s13244-022-01329-x)
Supplement: Supplementary file 1 — Additional file 1. An example of milestones for a sub-competency and the processing of missing data. [file 13244_2022_1329_MOESM1_ESM.pdf]

Table S1: A Supplemental Guide is available to provide the intent of each subcompetency, examples for each level, assessment methods or tools, and other available resources.

**Patients Care 1：Image Interpretation**				
Level 1	Level 2	Level 3	Level 4	Level 5
Identifies primary imaging findings	Identifies secondary and critical imaging findings and formulates differential diagnoses	Prioritizes differential diagnoses and recommends management options	Provides a single diagnosis with integration of current guidelines to recommend management, when appropriate	Demonstrates expertise and efficiency at a level expected of a subspecialist
Scores: 1 2	3 4	5 6	7 8	9
Comments：			Not Yet Completed Level 1 ☐	Not Yet Assessable ☐

Table S2: Missing rates of Each variable and management of missing data

Variables	Missing rate (%)	Management of missing data
Age	4 (0.1)	Markov Chain Monte Carlo (MCMC) method of multiple imputation to impute missing values and combine inferences by SAS9.4.
Working years	3 (0.1)	
Working hours per week	10 (0.3)	
Annual after-tax income	18 (0.5)	
Number of written radiology reports per day	14 (0.4)	

Note: A total of 3768 radiologists responded to our survey, 63 of them were not received SRT training during 2020 and 39 cases had large data deletion (more than 50% invalid data) and duplication. **3666 valid samples were included in this study.**
